# Disinfectant wipes transfer *Clostridioides difficile* spores from contaminated surfaces to uncontaminated surfaces during the disinfection process

**DOI:** 10.1186/s13756-020-00844-0

**Published:** 2020-11-04

**Authors:** Carine A. Nkemngong, Gurpreet K. Chaggar, Xiaobao Li, Peter J. Teska, Haley F. Oliver

**Affiliations:** 1 Department of Food Science, Purdue University, 745 Agriculture Mall Drive, West Lafayette, IN 47907 USA; 2 Diversey Inc., Charlotte, NC 28273 USA

**Keywords:** *Clostridioides difficile*, Cross-contamination, Disinfectants

## Abstract

**Background:**

Pre-wetted disinfectant wipes are increasingly being used in healthcare facilities to help address the risk of healthcare associated infections (HAIs). However, HAIs are still a major problem in the US with *Clostridioides difficile* being the most common cause, leading to approximately 12,800 deaths annually in the US. An underexplored risk when using disinfectant wipes is that they may cross-contaminate uncontaminated surfaces during the wiping process. The objective of this study was to determine the cross-contamination risk that pre-wetted disinfectant towelettes may pose when challenged with *C. difficile* spores. We hypothesized that although the tested disinfectant wipes had no sporicidal claims, they will reduce spore loads. We also hypothesized that hydrogen peroxide disinfectant towelettes would present a lower cross-contamination risk than quaternary ammonium products.

**Methods:**

We evaluated the risk of cross-contamination when disinfectant wipes are challenged with *C. difficile* ATCC 43598 spores on Formica surfaces. A disinfectant wipe was used to wipe a Formica sheet inoculated with *C. difficile*. After the wiping process, we determined log_10_ CFU on previously uncontaminated pre-determined distances from the inoculation point and on the used wipes.

**Results:**

We found that the disinfectant wipes transferred *C. difficile* spores from inoculated surfaces to previously uncontaminated surfaces. We also found that wipes physically removed *C. difficile* spores and that hydrogen peroxide disinfectants were more sporicidal than the quaternary ammonium disinfectants.

**Conclusion:**

Regardless of the product type, all disinfectant wipes had some sporicidal effect but transferred *C. difficile* spores from contaminated to otherwise previously uncontaminated surfaces. Disinfectant wipes retain *C. difficile* spores during and after the wiping process.

## Background

Pre-wetted disinfectant wipes are increasingly being used in healthcare facilities to disinfect equipment and environmental surfaces proximal to patients to reduce the risk of healthcare associated infections (HAIs) [1]. This may play a significant role in reducing the incidence of certain HAIs [2, 3]. However, despite efforts being made to reduce the incidence of HAIs, one out of 31 patients in the United States (US) acquires one or more HAIs on a daily basis [4]. Among pathogens implicated in the incidence of HAIs, *Clostridioides difficile* is among the most common in the US [5, 6]. The Center for Disease Control and Prevention (CDC) estimated that in 2017 there were approximately 223,900 hospitalized patients with *C. difficile* infections in the US with at least 12,800 deaths [6]. In acute care facilities, *C. difficile* infections result in approximately $4.8 billion in extra healthcare costs [5] due to prolonged hospital stays and readmissions [5, 7].

The contamination of environmental surfaces in healthcare facilities may account for up to 20% of HAIs [8]. Specifically, hard non-porous environmental surfaces such as bed rails [9–11] and bedside tables [9] may harbor *C. difficile* spores and contribute to transmission resulting in HAIs in healthcare facilities [12]. The eradication of *C. difficile* from environmental surfaces could be particularly challenging as spores can persist on environmental surfaces for months [8, 13]. Specifically, the persistence of *C. difficile* spores on environmental surfaces has been associated with the use of non-sporicidal cleaning agents such as quaternary ammonium compounds, which may increase *C. difficile* sporulation rates [14]. Consequently, the use of disinfectant wipes with *C. difficile* sporicidal claims have been recommended to reduce the incidence of HAIs [15], as wipes have been suggested to increase compliance with standard cleaning and disinfection protocols [16].

In the US, healthcare facilities commonly use visual evaluations to determine “contamination levels” on hard non-porous surfaces prior to disinfection [17]. Consequently, irrespective of spore or vegetative state on surfaces, broad-spectrum antimicrobial wipes, such as those loaded with quaternary ammonium compounds, are relied on for routine disinfection practices [18–20]. However, pre-wetted disinfectant wipes may pose the risk of cross-contaminating “clean” surfaces during the wiping process [21]. This may be product-dependent as Siani et al. demonstrated that sodium hypochlorite wipes are more sporicidal against *C. difficile* than quaternary ammonium compounds (QAC) [1]. Other differences in the bactericidal efficacy of disinfectant active ingredients have been reported by Lineback et al. who demonstrated that hydrogen peroxides and sodium hypochlorites were more bactericidal against bacterial biofilms than QAC [22]. In addition, the Environmental Protection Agency (EPA) has no recommendation on the maximum surface area that could be disinfected with a towelette in order to optimize bactericidal efficacy, while minimizing the risk of cross-contaminating low risk surfaces.

The risk of pathogen transmission by the hands of healthcare workers and patients has been widely investigated [23–25]. However, less work [17, 26] has been done to determine the risk of cross-contamination by disinfectant wipes using real world techniques in vitro as standard testing for the registration of towelette products rarely mandate the simulation of real world wiping scenarios [3]. The objective of this study was to determine the cross-contamination risk that disinfectant towelettes with no sporicidal claims may pose when challenged with *C. difficile* spores. We hypothesized that although the tested disinfectant wipes have no sporicidal claims, they will reduce *C. difficile* spore loads, but cross-contamination may still occur. On a related note, we hypothesized that towelettes with sporicidal claims will present a lower cross-contamination risk than wipes without sporicidal claims. We also hypothesized that compared to quaternary ammonium products, hydrogen peroxide disinfectant towelettes will present a lower risk of cross-contaminating low risk surfaces after wiping down an area inoculated with *C. difficile* spores.

## Methods

### Disinfectants and bacterial strain used in this study

This study investigated the risk of cross-contamination of seven disinfectant towelette products; six with non-sporicidal claims and one product with sporicidal claims (Table 1). Ready-to-use wipes containing 1.312% sodium hypochlorite with an EPA registered sporicidal claim were used as a control. *C. difficile *spores ATCC 43598 were produced following EPA MLB SOP-MB-28 [27] and used to study the risk of cross-contamination by disinfectant wipes following a modified version of EPA MLB SOP-MB-31 [28].

**Table 1:** Active ingredients and contact times for disinfectant towelettes tested in this study

Disinfectant product ^a^	Disinfectant active ingredient(s)^c^	Dilution at use	Active level at use^e^	Label contact time (mins)^f^
SH^b^	1.312% sodium hypochlorite	RTU^d^	1.25%	4
HP1	1.4% hydrogen peroxide	RTU	1.4%	1
HP2	0.5% hydrogen peroxide	RTU	0.5%	1
HP3	0.5% hydrogen peroxide	RTU	0.5%	1
QA1	0.25% n-alkyl (68%C_12_, 32%C_14_) dimethylethylbenzyl ammonium chloride 0.25% n-alkyl (60% C_14_, 30% C_16_, 5% C_12_, 5% C_18_) dimethyl benzyl ammonium chloride 55% isopropanol	RTU	0.5%^g^ + 55%	2
QA2	0.76% didecyldimethyl ammonium chloride 15% isopropanol 7.50% ethanol	RTU	0.76%^g^ + 22.5%	1
QA3	0.233% disobutylphenolxyethoxyethyl dimethyl benzyl ammonium chloride 14.3% isopropanol	RTU	0.233%^g^ + 14.3%	2

^a^Abbreviated naming scheme for commercially available EPA registered disinfectants used in this study

^b^Control disinfectant with *C. difficile* claim

^c^Active ingredients concentration

^d^Ready-to-use

^e^Active ingredient concentration after dilution

^f^Defined label contact time

^g^Total quaternary ammonium plus alcohol content

### Test surface sterilization, inoculation and disinfection

**Fig. 1:**
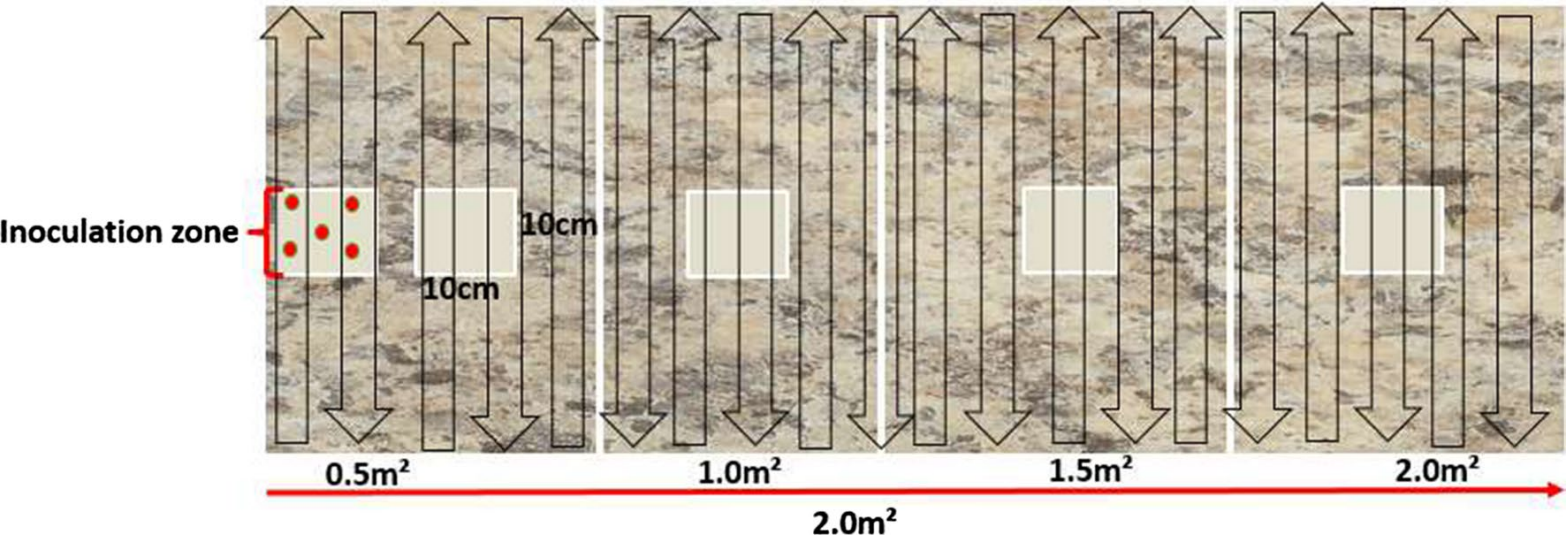
Schematic diagram of the Formica surface used for wipe testing. Two meters of Formica were delineated into 0.5 m^2^ sections. 5.0 × 10^8^ log_10_CFU *C. difficile* spores were spotted onto the inoculation zone (i-zone) as represented by red dots. The entire surface was wiped in an up and down motion across the entire surface as indicated by black outlined arrows from left to right. 10 cm × 10 cm (100 cm^2^) sampling zones (light gray squares) were sampled to recover potentially cross-contaminated spores

A two-meter square area of Formica sheeting was marked into different lengths and labeled as follows: inoculation zone (i-zone), 0.5 m^2^, 1 m^2^, 1.5 m^2 ^and 2 m^2^ (Fig. 1). For the i-zone and for every 0.5 m^2^ area, a 10 cm × 10 cm (100 cm^2^) area was marked in the center of the defined lengths to recover spores from the surface. The entire Formica surface was sterilized by a three-step process. Progressively, the surface was cleaned with 7.0% hydrogen peroxide, 10% bleach and 70% ethanol. Following each of the first two disinfection processes, three rinses each with 250 ml of sterile distilled water was used to rinse the surface. This was followed by a final application of 70% ethanol. The Formica sheet was left to air-dry on a clean laboratory bench.

The *C. difficile* spore inoculum was prepared following EPA MLB SOP-MB-31 [28] and used to test the risk of cross-contamination by disinfectant wipes from the “i-zone” to other portions of the Formica sheet. A final spore suspension of 500 µL was prepared with a soil load composed of 25 µL 0.05% bovine serum albumin (BSA; Fisher bioreagents, Ottawa, Canada), 35 µL 0.05% yeast extract (ACROS Organics, New Jersey, US), 100 µL 0.004% mucin stock (Abnova, Walnut, USA), and 340 µL *C. difficile* spores (prepared following EPA MLB SOP MB-28; stored at − 20 ± 5 °C). After sterilizing the entire two-meter square area, a marked 10 cm × 10 cm (100 cm^2^) area in the i-zone was inoculated with five 10 µl aliquots of the *C. difficile* spore suspension (approximately 5.0 × 10^8^ colony forming units per ml) following EPA MLB SOP-MB-28 [29]. The first two towelettes from each disinfectant were discarded and the third used for testing to ensure enough disinfectant liquid load on the towelettes. This was used to wipe the entire two-meter square Formica sheet from the i-zone of the Formica to the two-meter mark of the Formica. The surface was wiped in a continuous up and down movement (Fig. 1). This was repeated for the entire surface area of the Formica sheet starting with the i-zone. The designated surfaces were left at room temperature for the disinfectants’ defined label contact times (Table 1). At the full contact time, swab samples of 100 cm^2^ were collected from every 0.5 m^2^ starting with the inoculation area using PUR-Blue Swabs (World BioProducts, Libertyville, IL; containing 10 mL sterile HiCap neutralizing buffer). The swab samplers were each vortexed for 30 s to release the bacterial spores from the sponge into the solution. The used wipes were placed in a sterile stomacher bag (Whirl–Pak, Nasco, Fort Atkinson, WI) containing 50 mL of 0.52% neutralizing buffer (BD Difco, Becton, Dickinson and Company, MD, USA), shaken for five min at 230 rpm using a stomacher to detect viable *C. difficile* spores on the towelette. Ten ml neutralizing buffer from the PUR-Blue swabs and the sterile stomacher bags were vacuum-filtered onto a membrane filter (0.2 μm pore size, 47 mm grid, individual sterile pack; Pall Corporation, Port Washington, NY) following EPA MLB SOP-MB-31 [28]. The membrane filters were aseptically transferred to pre-reduced brain–heart infusion agar with yeast extract, horse blood and sodium taurocholate plates (BHIY-HT; Anaerobe Systems, Morgan Hill, CA) and incubated under anaerobic conditions at 36 ± 1 °C for 120 ± 4 h before colonies characteristic of *C. difficile* spores as stated in EPA-MLB SOP-MB-31 were counted. Anaerobic conditions were achieved using an anaerobic jar (BD BBL GasPak, Becton, Dickinson and Company, Franklin Lakes, NJ) and CO_2_ gas generating packs (BD GasPak, Becton, Dickinson and Company, MD, USA). Five biological replicates were conducted for each of the disinfectant products tested and one technical replicate performed for each biological replicate per surface area tested.

### Statistical analysis

*C. difficile* spores were recovered from five test zones of a two-meter square Formica sheet and from used disinfectant wipes; counts were log_10_–transformed. Average log_10_ CFU were calculated for wipes and defined sampled surfaces to test for statistically significant differences among eight disinfectant products. Specifically, we tested for differences among sampled surfaces by analyzing log_10_ CFU/100 cm^2^ counts recovered after disinfection. We also analyzed log_10_ CFU/wipe used to test for the risk of cross-contamination from the i-zone to low risk surfaces. The least squares method of the Proc Glimmix test was used to fit liner models (n = 42, α = 0.05) and to test for interactions between disinfectant log_10_CFU/100 cm^2^ and the surface area sampled. Surface area wiped and product type were treated as variables with continuous effects (repeated measures in Proc Glimmix). Tukey adjustments were used to test for significant differences in mean log_10_ CFU among disinfectant products. The same procedure was also used to test for significant differences among surfaces treated with the same disinfectant wipe. All statistical tests were conducted using SAS version 9.4 (SAS institute, Cary, NC).

## Results

### Disinfectant wipes transfer varied levels of *C. difficile* spores to low risk (not previously contaminated) hard non-porous surfaces

**Fig. 2:**
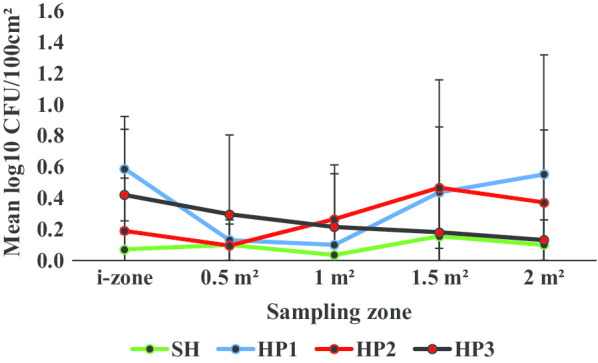
Mean log_10_ CFU/100 cm^2^ remaining on sampled portions of the Formica sheet post disinfection with SH or hydrogen peroxide disinfectant wipes

**Fig. 3:**
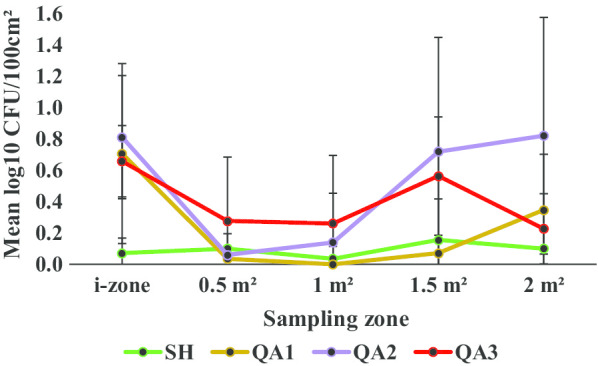
Mean log_10_ CFU/100 cm^2^ remaining on sampled portions of the Formica sheet post disinfection with SH or quaternary ammonium alcohol disinfectant wipes

Regardless of disinfectant product, both the sporicidal or non-sporicidal disinfectant wipes cross-contaminated low risk or otherwise previously uncontaminated surfaces from the i-zone (Figs. 2, 3**).** On average, 0.49 ± 0.27 log_10_ CFU/100 cm^2^ was recovered from the i-zone after the wiping process. Overall, a disinfectant wipe transferred a mean of 0.13 ± 0.12 and 0.34 ± 0.27 log_10_ CFU/100 cm^2^ from the i-zone to the 0.5 m^2^ and 2.0 m^2^ risk surfaces respectively. Similarly, each wipe transferred on average, 0.13 ± 0.11 and 0.36 ± 0.25 log_10_ CFU/100 cm^2^ from the i-zone to the 1 m^2^ and 1.5 m^2^ surfaces respectively.

The surface area wiped was statistically significant (*P* < 0.05). On average, the log_10_ CFU/100 cm^2^ transferred to 0.5 m^2^ and 1 m^2^ surfaces from the i-zone were significantly lower compared to the log_10_ CFU/100 cm^2^ recovered from the i-zone post disinfection (*P* < 0.05). However, there were no statistically significant differences among the *C. difficile* spore log_10_ CFU/100 cm^2^ transferred to the 1.5 m^2^ and 2.0 m^2^ surfaces and the log_10_ CFU/100 cm^2^ detected from the i-zone after the wiping process (*P* ≥ 0.05). There were also no statistically significant differences among the mean log_10_ CFU/100 cm^2^ detected from the 0.5 m^2^, 1 m^2^, 1.5 m^2^, and 2 m^2^surfaces post disinfection (*P* ≥ 0.05).

Regarding cross-contaminating otherwise low risk surfaces (0.5–2.0 m^2^), the product type was statistically relevant (*P* < 0.05). From the i-zone, the mean log_10_ CFU/100 cm^2^ transferred to the 0.5–2.0 m^2^ zones ranged from 0.04 ± 0.05 (SH), 0.11 ± 0.16 (QA1), 0.21 ± 0.07 (HP3), 0.28 ± 0.26 (HP1), 0.30 ± 0.16 (HP2), 0.33 ± 0.16 (QA3) and 0.43 ± 0.39 (QA2). QA2 and QA3 wipes transferred at significantly higher log_10_ CFU/100 cm^2^ from the i-zone to the 0.5 m^2^, 1 m^2^, 1.5 m^2^, and 2 m^2^ surfaces compared to the control, SH (*P* < 0.05; Fig. 3; Additional file 1). However, QA1 transferred significantly lower log_10_ CFU/100 cm^2^ from the i-zone to the 0.5–2.0 m^2^ areas than QA2 and QA3 (*P* < 0.05; Fig. 3; Additional file 1). There were no statistically significant differences in the cross-contamination levels (mean log_10_ CFU/100 cm^2^) among SH, HP1, HP2, HP3, and QA1 (*P* ≥ 0.05; Figs. 2, 3; Additional file 1).

### High levels of *C. difficile* spores were recovered from disinfectant towelettes after use

**Fig. 4:**
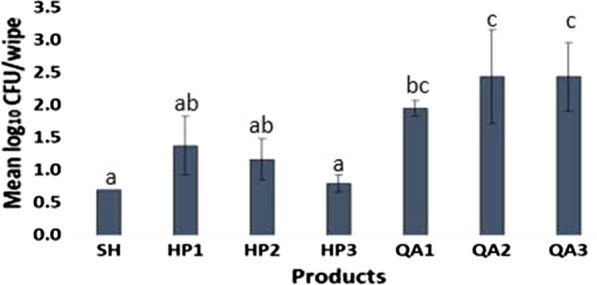
Mean log_10_ CFU remaining on used wipes post disinfection with SH, hydrogen peroxide or quaternary ammonium alcohol disinfectant wipes. Bars with the same Turkey letter are statistically similar

Overall, all disinfectant wipes retained *C. difficile* spores after surface disinfection (Fig. 3); there were statistically significant differences among products (*P* < 0.05). The log_10_ CFU/wipe ranged from 0.70 ± 0.00 (minimum detection level) for SH to 2.43 ± 0.72 for QA2 after use **(**Fig. 3**)**. The mean log10 CFU/wipe for the quaternary alcohol products were 1.95 ± 0.12, 2.43 ± 0.72 and 2.43 ± 0.52 for QA1, QA2 and QA3, respectively. After the wiping process, control wipes (SH) were significantly less contaminated than towelettes of QA1, QA2 and QA3 (*P* < 0.05; Fig. 4). Comparing QA products only, there were no statistically significant differences among the log_10_ CFU/wipe recovered on wipes of QA1, QA2 and QA3 post disinfection (*P* ≥ 0.05; Fig. 3). Similarly, there were no significant differences among the log_10_ spore counts on HP1, HP2 and HP3 towelettes after the wiping process as the average log10 CFU/wipe were 1.38 ± 0.45, 1.17 ± 0.31 and 0.79 ± 0.14 for HP1, HP2 and HP3, respectively (*P* ≥ 0.05; Fig. 4**)**. Also, there were no statistically significant differences between log_10_ CFU counts from SH and HP1, and HP2 and HP3 wipes **(P** ≥ 0.05; Fig. 4**)**.

In comparing the log_10_ CFU/wipe of QA and HP products, there were no statistically significant differences among log_10_ CFU/wipe recovered from HP1, HP2 and QA1 wipes (*P* ≥ 0.05; Fig. 4). However, significantly lower contamination levels were observed on used HP1, HP2, and HP3 wipes compared to QA2 and QA3 **(P** < 0.05; Fig. 4).

### The sporicidal efficacy of disinfectant towelettes varies by product type and surface area

The product type was statistically significant (*P* < 0.05). The mean log_10_ CFU/100 cm^2^ recovered from the i-zone—2.0 m^2^ post disinfection ranged from 0.05 ± 0.04 for SH to 0.51 ± 0.38 for QA2. For the QA products, the mean log_10_ CFU/100 cm^2^ (i-zone—2.0 m^2^) were 0.23 ± 0.30 (QA1), 0.51 ± 0.38 (QA2) and 0.40 ± 0.20 (QA3). For HP products, the average log_10_ CFU/100 cm^2^ recovered from the i-zone—2.0 m^2^ areas were 0.34 ± 0.26 (HP1), 0.28 ± 0.15 (HP2) and 0.25 ± 0.11 (HP3). SH was significantly more sporicidal than QA2 and QA3 (*P* < 0.05) as on average, lower log_10_ CFU/100 cm^2^ were recovered from the i-zone and subsequent surfaces (0.5 m^2^, 1 m^2^, 1.5 m^2^, and 2 m^2^) (Fig. 3; Additional file 1). However, there were no statistically significant differences among the sporicidal efficacies of SH, HP1, HP2, HP3 (Fig. 2; Additional file 1) and QA1 (*P* ≥ 0.05; Fig. 3, Additional file 1) as the mean log_10_ CFU/100 cm^2^ across the tested surfaces were very similar.

Surface area wiped was statistically significant (*P* < 0.05) and overall, the sporicidal efficacy of disinfectant wipes decreased with an increase in the surface area wiped from 0.5 to 2 m^2^ (Figs. 2, 3; Additional file 1). Compared to the 1.5 m^2^ and 2 m^2^ areas, disinfectant towelettes were statistically more sporicidal when used on the 0.5 m^2^ and 1 m^2^ surface areas (*P* < 0.05). This was the case as log_10_ CFU/100 cm^2^ from the 0.5 m^2^ and 1 m^2^ surfaces were significantly lower relative to the i-zone (*P* < 0.05; Figs. 2, 3). Overall, there were no statistically significant differences in the sporicidal efficacy of disinfectant towelettes when the log_10_CFU/100 cm^2^ from the i-zone, 1.5 m^2^ and 2 m^2^ areas were compared (*P* ≥ 0.05). Similarly, regardless of the active ingredient class, and without comparison to the i-zone, there were no statistically significant differences in the sporicidal efficacies recorded within the 0.5 m^2^, 1 m^2^, 1.5 m^2^ and 2 m^2^ surface areas (*P* ≥ 0.05, Figs. 2, 3; Additional file 1).

## Discussion

In this study, we determined the cross-contamination risk that disinfectant wipes may pose during and after the wiping process. We established that during the wiping process, disinfectant wipes transfer *C. difficile* spores from a contaminated surface (i-zone) to otherwise uncontaminated surfaces during the disinfection process. We also found that among all the used disinfectant wipes tested in this study, viable *C. difficile* spores were detected on the wipes post disinfection. Overall, we found that after the wiping process, the log_10_ CFU/100 cm^2^ detected from the 0.5 m^2^ and 1 m^2^ surfaces were significantly lower compared to those recovered from the i-zone. However, there were no significant differences among the log_10_ CFU/ 100 cm^2^ transferred to the 1.5 m^2^ and 2.0 m^2^ surfaces and the log_10_ CFU/100 cm^2^ recovered from the i-zone post-disinfection.

### Disinfectant wipes cross-contaminate hard non-porous surfaces

Cross-contamination is described by the CDC as the transfer of bacteria by contact from one surface to another [30]. Disinfectant wipes were the transfer “agents” between the surface inoculated with *C. difficile* spores and non-contaminated surfaces. In a similar study, Lopez et al. found that *Bacillus thuringiensis* spores inoculated on inanimate surfaces were transferred from wipe-disinfected fomites to fingers [31]. More recently, Becker et al. demonstrated that disinfectant wipes loaded with propanol or quaternary ammonium compounds (QAC) transferred viruses from a 25 cm^2^ inoculated surface onto three other surfaces of the same size in the process of using the wipes [26].

Compared to the i-zone, the log_10_CFU/100 cm^2^ from the 0.5 m^2^ and 1 m^2^ low risk surfaces were significantly lower than the log_10_CFU/100 cm^2^ of the i-zone post disinfection. This could be explained by the observation that more disinfectant liquid was released from the wipe onto the 0.5 m^2^ and 1 m^2^ areas compared to the 1.5 m^2^ and 2 m^2^ areas. This was evident as the 0.5 m^2^ and 1 m^2^ surfaces were visibly wet after the wiping process. While the i-zone was also visibly wet, it is likely that the amount of liquid disinfectant dispensed from products without sporicidal claims were insufficient to result in any significant kill. This may be the case as within the i-zone, wipes were challenged with approximately 5.0 × 10^8^ log_10_ CFU, and lower log_10_ CFU thereafter as it is likely that disinfectant wipes did not physically pick up all 5.0 × 10^8^ log_10_ CFU from the i-zone. Moreover, West et al. suggested that the high amount of disinfectant liquid released from a quaternary ammonium disinfectant wipe for example may cause a “gliding” effect resulting in a reduction in the physical removal of microorganisms by wipes [32]. In a previous study by our group [32], we found that the percent of liquid released per 0.1 m^2^ of a Formica surface significantly decreased as the surface area wiped increased. We found that overall, the log_10_ CFU/100 cm^2^ recovered from the 1.5 m^2^ and 2.0 m^2^ areas were comparable to that recovered from the i-zone post disinfection. This suggests that in the disinfection of larger surfaces, cross-contamination may continuously increase to levels that are comparable with the “residual” spores from the contamination source post disinfection.

Regardless of the product type, and without comparison to the i-zone, there were no significant differences in the log_10_ CFU/100 cm^2^ of spores detected from 0.5 m^2^, 1.0 m^2^, 1.5 m^2^ and 2.0 m^2^ surface areas. In a similar study, Becker et al. did not find significant differences in the titer of viruses detected from three 25 cm^2^ uncontaminated surfaces after wiping them with a QAC disinfectant wipe previously used on a contaminated surface [26]. The reported risk of cross-contamination by *C. difficile* spores is particularly relevant in healthcare settings as *C. difficile* infections have been associated with contaminated environmental surfaces and are a leading cause of HAIs in the US [6, 33–35].

Among disinfectants with the same active ingredients a (e.g. QA products) there were significant differences in the cross-contamination risk as QA1 presented a lower cross-contamination risk than QA2 and QA3. It is possible that the higher alcohol content of QA1 (55%) compared to 22.5% for QA2 and 14.3% for QA3 made it more sporicidal (Table 1). This could be further supported as the spore load on QA1 wipes after use were similar to those of products with active ingredient classes that are generally sporicidal in nature (HP1 and HP2) [36]. There were no statistically significant differences among the cross-contamination risk of HP products and SH. This suggest that although these products did not carry sporicidal claims, the products exhibited significant sporicidal action. This could be explained by the fact that hydrogen peroxides are generally a class of active ingredients used for sterilization purposes [36] owing to their ability to inactivate spores.

### Used disinfectant wipes are potential cross-contamination agents after use

Although used disinfectant wipes are typically considered standard medical waste [37], we found that used disinfectant wipes may retain high numbers of *C. difficile* spores after use. This demonstrates a “mechanical” spore removal mechanism from contaminated surfaces during the wiping process. This finding is similar to those of Gonzales et al. who reported a physical removal of *Clostridium sporogenes* and *Bacillus atrophaeus* spores by antimicrobial wipes during the wiping process [38]. Kenters et al. also reported a similar mechanical removal effect by wipes challenged with *C. difficile* spores [39]. We observed that after wiping down the Formica sheet, all the used disinfectant towelettes were dry to the feel. This suggests that after use, the residual disinfectant liquid on the wipe may be insufficient to kill the spores picked up on the cloth within the product’s label-defined contact time. The ability for disinfectant wipes to retain spores after use may present a considerable cross-contamination risk especially if the same wipe is used on multiple surfaces or pieces of equipment. Siani et al. reported that disinfectant towelettes with no sporicidal claims that harbored *C. difficile* spores could eventually serve as cross-contamination agents [1].

The wipe design and substrate may also play a significant role in the level of organisms removed by the wipe [40]. Although the specific effects of the wipe materials were not evaluated in this study, differences in the levels of spores retained on the wipe could be associated with the wipe material type and with the amount of disinfectant liquid loaded on the wipe. Some wipe material types may hold more disinfectant liquid, which may be helpful in disinfection. We observed that wipes that had a rough feel probably due to their low cotton content (mostly the QA wipes) retained higher spore loads. In a 2012 study, Masuku et al. reported that the kind of material the wipe was made of, significantly impacted disinfection levels [41].

### Hydrogen peroxide-based wipes are more sporicidal than wipes with QACs

All hydrogen peroxide-based disinfectants tested in this study were more sporicidal than most (2/3) of the QAC disinfectant wipes tested. The sporicidal activity of hydrogen peroxides has been associated with their ability to produce free hydroxyl radicals after binding to deoxyribonucleic acids (DNA) [42]. These hydroxyl free radicals damage DNA and cell membrane lipids [42]**.** Although the tested disinfectants, with the exception of SH, had no sporicidal claims, we found that all the tested disinfectants reduced spore loads. This is likely a joint effect of physical spore removal by the wipe substrate and spore inactivation by the disinfectants [3, 39, 43]. Specifically, Rutala et al. reported that disinfectant wipes with no sporicidal claims had sporicidal effects, and the wipes could physically remove more than 2.9 logs of *C. difficile* spores from inoculated surfaces [43]. Our findings were similar to that of Rutala et al. [43] as the sporicidal efficacy of disinfectant wipes with non-sporicidal claims (HP1, HP2, HP3 and QA1) were comparable to that of SH with a sporicidal claim.

The US EPA requirements for obtaining a disinfectant label claim for *C. difficile* require a minimum of a six log_10_ reduction [44]. But in our study, we found no statistical difference in disinfectant performance between all of the hydrogen peroxide wipes without a *C. difficile* sporicidal label claim and the sodium hypochlorite-based product with a *C. difficile* sporicidal label claim. This suggest that the benefits in efficacy in passing the EPA method may not translate to actual differences in efficacy in real world use, as simulated in this study. Thus, there may be no actual clinical benefit from using a sporicidal disinfectant wipe in reducing patient risk of *C. difficile* infection versus using a hydrogen peroxide (non-sporicidal) disinfectant wipe. This needs further study.

We acknowledge that our study is limited as we did not investigate the effect the different wipe materials could have on the risk of cross-contamination. We also did not study the impact of a prolonged contact time on the inactivation of spores retained by used disinfectant wipes. It may also be of interest to investigate the cross-contamination risk presented by traditional “bucket and wipe” disinfection methods in comparison to the use of ready-to-use disinfectant wipes; these study limitations warrant further study.

## Conclusion

Overall, disinfectant wipes may transfer *C. difficile* spores from contaminated to uncontaminated surfaces and retain high spore loads after the disinfection process, but the rate at which this occurs varies by product and likely is affected by the disinfectant liquid load, chemistry, and wiping material. We determined that non-sporicidal wipes reduce spore load, but the need to conduct similar studies using prevalent HAIs pathogens as *Staphylococcus aureus* and *Pseudomonas aeruginosa* remains. We definitively established that when disinfectant wipes are used on large surface areas, they may present a considerable cross-contamination risk, which could put patients at greater risk of HAIs.

## Supplementary information

**Additional file 1**. Supplemental Data outlines the mean log_10_ CFU/100 cm^2^ obtained from the i-zone, 0.5 m^2^, 1.0 m^2^, 1.5 m^2^ and 2.0 m^2^ surface areas for all evaluated products.

## Data Availability

All quantitative data generated or analysed during this study are included in this published article.

## Abbreviations

ATCC: American type culture collection
BHIY-HT: Brain-heart infusion agar with yeast extract, horse blood and sodium taurocholate
CDC: Center for Disease Control
CFU: Colony forming unit
DNA: Deoxyribonucleic acids
EPA: Environmental Protection Agency
HAIs: Healthcare-associated infections
TSB: Tryptic soy broth
QAC: Quaternary ammonium compounds
