# Supplementary material for: Disinfectant wipes transfer Clostridioides difficile spores from contaminated surfaces to uncontaminated surfaces during the disinfection process
Source: Antimicrob Resist Infect Control. 2020 Nov 4;9:176. doi: 10.1186/s13756-020-00844-0 (PMC7641809; doi:10.1186/s13756-020-00844-0)
Supplement: Supplementary file 1 — Additional file 1. Supplemental Data outlines the mean log10 CFU/100 cm2 obtained from the i-zone, 0.5 m2, 1.0 m2, 1.5 m2 and 2.0 m2 surface areas for all evaluated products. [file 13756_2020_844_MOESM1_ESM.pdf]

Supplemental Data

**Product name**	**Sampling zone**	**Log_{10} CFU/100 cm^2**
SH	i-zone	0.07 ± 0.10
	0.5m^2	0.00 ± 0.00
	1.0m^2	0.04 ± 0.08
	1.5m^2	0.16 ± 0.26
	2.0m^2	0.00 ± 0.00
QA1	i-zone	0.71 ± 0.57
	0.5m^2	0.04 ± 0.08
	1.0m^2	0.00 ± 0.00
	1.5m^2	0.07 ± 0.10
	2.0m^2	0.35 ± 0.36
QA2	i-zone	0.81 ± 0.39
	0.5m^2	0.06 ± 0.13
	1.0m^2	0.14 ± 0.31
	1.5m^2	0.72 ± 0.73
	2.0m^2	0.82 ± 0.75
QA3	i-zone	0.66 ± 0.23
	0.5m^2	0.28 ± 0.41
	1.0m^2	0.26 ± 0.43
	1.5m^2	0.56 ± 0.38
	2.0m^2	0.23 ± 0.22
HP1	i-zone	0.59 ± 0.33
	0.5m^2	0.13 ± 0.13
	1.0m^2	0.00 ± 0.00
	1.5m^2	0.44 ± 0.72
	2.0m^2	0.55 ± 0.77
HP2	i-zone	0.19 ± 0.34
	0.5m	0.10 ± 0.14
	1.0m^2	0.26 ± 0.35
	1.5m^2	0.47 ± 0.39
	2.0m^2	0.37 ± 0.47
HP3	i-zone	0.42 ± 0.42
	0.5m	0.30 ± 0.51
	1.0m^2	0.22 ± 0.34
	1.5m^2	0.18 ± 0.27
	2.0m^2	0.13 ± 0.13
